# Transgelin promotes lung cancer progression via activation of cancer-associated fibroblasts with enhanced IL-6 release

**DOI:** 10.1038/s41389-023-00463-5

**Published:** 2023-03-29

**Authors:** Chanjun Sun, Kaishang Zhang, Chen Ni, Jiajia Wan, Xixi Duan, Xiaohan Lou, Xiaohan Yao, Xiangnan Li, Ming Wang, Zhuoyu Gu, Pengyuan Yang, Zhenzhen Li, Zhihai Qin

**Affiliations:** 1 Medical Research Center, The First Affiliated Hospital of Zhengzhou University, Zhengzhou University, Zhengzhou, 450052 Henan China; 2 Thoracic Surgery Department, The First Affiliated Hospital of Zhengzhou University, Zhengzhou University, Zhengzhou, 450052 Henan China; 3 Key Laboratory of Infection and Immunity of CAS, CAS Center for Excellence in Biomacromolecules, Institute of Biophysics, Chinese Academy of Sciences, University of Chinese Academy of Sciences, 100101 Beijing, China; 4 Key Laboratory of Protein and Peptide Pharmaceuticals, Institute of Biophysics, Chinese Academy of Sciences, No. 15 Datun Road, Chaoyang Area, 100101 Beijing, China

**Keywords:** Cancer microenvironment, Non-small-cell lung cancer

## Abstract

Cancer-associated fibroblasts (CAFs), the principal constituent of the heterogenous tumor microenvironment, have been shown to promote tumor progression; however, the underlying mechanism is still less clear. Here, we find that transgelin (TAGLN) protein levels increased in primary CAFs isolated from human lung cancer, compared with those in paired normal fibroblasts. Tumor microarrays (TMAs) revealed that increased stromal TAGLN levels correlates with more lymphatic metastasis of tumor cells. In a subcutaneous tumor transplantation model, overexpression of *Tagln* in fibroblasts also increased tumor cell spread in mice. Further experiments show that *Tagln* overexpression promoted fibroblast activation and mobility in vitro. And TAGLN facilitates p-p65 entry into the nucleus, thereby activating the NF-κB signaling pathway in fibroblasts. Activated fibroblasts promote lung cancer progression via enhancing the release of pro-inflammatory cytokines, especially interleukine-6 (IL-6). Our study revealed that the high levels of stromal TAGLN is a predictive risk factor for patients with lung cancer. Targeting stromal TAGLN may present an alternative therapeutic strategy against lung cancer progression.

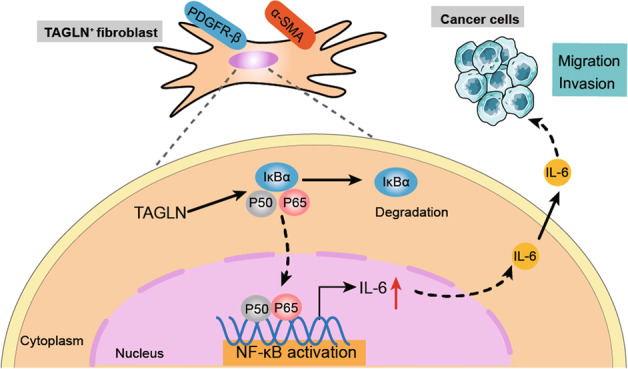

## Introduction

Lung cancer is the most common cause of cancer-related death worldwide, accounting for ~27% of all cancer-related deaths annually [1]. The overall survival rate for patients with lung cancer remains unsatisfactory; less than 7% survive more than 10 years after diagnosis, independent of the cancer stage [2]. Current treatments and therapies are insufficient to reduce this mortality rate. Diagnosis at an advanced stage and lack of effective and personalized medicine reflect the need for a better understanding of the mechanisms underlying lung cancer progression. Therefore, it is particularly important to discover new markers for the early diagnosis of lung cancer.

Cancers are composed of cancer cells and many types of non-cancerous cells, including cancer-associated fibroblasts (CAFs), cancer-associated macrophages, and lymphocytes. These cells, together with the tumor vasculature and extracellular matrix, constitute the tumor microenvironment (TME). The TME is critical not only for cancer development and progression but also for tumor immunity and chemotherapy resistance [3]. CAFs are often the most abundant stromal cells and play a crucial and complex role in cancer development. Although the pro- or anti-tumor effects of CAFs remain controversial, it is generally accepted that CAFs can promote tumor growth [4], disease progression [5], and chemotherapy resistance [6]. Moreover, CAFs have been shown to increase tumor aggressiveness (survival, invasion, and chemoresistance) by secreting soluble factors, including cytokines, growth factors, and chemokines [7–9].

Transgelin (TAGLN), also called SM22 and first identified in 1987, is an actin-binding protein that belongs to the calponin family [10]. TAGLN promotes the aggregation of G-actin to F-actin by regulating actin cytoskeleton dynamics [11]. Zhong et al. found that activated TAGLN-actin could modulate the cytoskeleton and promote cell contraction [12]. As an actin crosslinking protein, TAGLN participates in cell movement by improving the formation of podosomes and several biological processes related to cancer progression, such as differentiation, proliferation, migration/invasion, and apoptosis. Chen et al. found that in bladder cancer, TAGLN is highly expressed and correlated with prognosis [13]. Recently, TAGLN was reported to be a poor prognostic factor in advanced stage colorectal cancer, promoting tumor growth and metastasis [14]. Increased levels of TAGLN have also been associated to poor prognosis and metastasis in other types of cancer, such as esophageal [15, 16], pancreatic [17, 18], lung [19] and colorectal cancers [20].

Importantly, TAGLN is expressed not only in epithelial cells but also in several different cell types such as fibroblasts, endothelial cells, and immune cells [21]. Recent studies highlighted the TAGLN functions in fibroblasts and their crosstalk with cancer cells. Stromal TAGLN levels are enhanced during gastric cancer progression and related to tumor metastasis through increased matrix metalloproteinase-2 signaling [22]. Rho et al. observed that TAGLN upregulation was strictly localized to the tumor-induced reactive myofibroblastic stromal tissue compartment in human lung adenocarcinoma tissue [23]. TAGLN has also been identified as a fibroblast-specific biomarker of poor prognosis in colorectal cancer (CRC) in a single-cell multiomics sequencing study, with 21 patients with CRC and 6 cancer-free individuals [24]. Elsafadi et al. evaluated 275 tumor and 349 non-tumor tissues for TAGLN expression using the TCGA/GTEx COAD dataset [14]. Though TAGLN was found to be downregulated in CRC, increased TAGLN levels were associated with advanced CRC stages and correlated with a poor overall survival and disease-free survival [14]. Despite these studies, whether TAGLN promotes development of CAF phenotype in normal fibroblasts (NFs) and the mechanism by which TAGLN-positive CAFs modulate tumor, especially lung cancer, progression remain largely unclear.

Here, we show that TAGLN overexpression can promote tumor spreading and tumor cell migration/invasion through the release of pro-inflammatory cytokines, namely interleukin-6 (IL-6), via NF-κB signaling pathway activation. Targeting TAGLN in CAFs may be a promising strategy for lung cancer therapy.

## Materials and methods

### Patient samples

Tumors and adjacent normal tissues (at least 5 cm from the tumor), resected surgically from patients with lung cancer, were obtained from the Department of Thoracic Surgery of the First Affiliated Hospital of Zhengzhou University (Zhengzhou, China). Lung cancer samples were processed for CAF isolation after informed consent was obtained, in accordance with the Declaration of Helsinki. This study was approved by the Ethics Committee of the First Affiliated Hospital of Zhengzhou University.

### EGFR-driven spontaneous lung cancer model

CCSP rtTA/EGFR^L858R^ (C/L858R) mice, a previously described mouse model expressing the mutant EGFR^L858R^ in type II pneumocytes [25], were obtained from Professor Lin Xi of Tsinghua University. For the induction of lung tumor formation, doxycycline (1 mg/ml) was administered in drinking water to 5-week-old mice, for 3 months.

### Subcutaneous transplantation tumor mouse model: lung cancer cells and fibroblasts syngeneic and orthotopic co-grafting

For the subcutaneous tumor model, a 1:3 mixture of LLC cells (5 × 10^5^) and fibroblasts (1.5 × 10^6^) contained in 100 μl of PBS was injected subcutaneously into the back of anesthetized (2% isoflurane, RWD) 8-week-old female C57BL/6N mice (Vital River Laboratories, Beijing, China). For the fibroblasts, mouse cancer-associated fibroblasts (mCAFs) and mouse normal fibroblasts (mNFs) were used in the first animal experiment, and *Tagln*-overexpressing (*Tagln*^*OE*^) or *Tagln*-knockdown (*Tagln*^*sh*^) fibroblasts were used in the second experiment. Each group consisted of at least five mice. Tumor length (L) and width (W) were measured every other day, starting on day 6 (after injection). The tumor volume was calculated using the following formula: LW^2^/2. Twenty-one days after injection, mice were euthanized, and the primary subcutaneous tumors and lungs were removed, analyzed, and paraffin-embedded before slicing and staining.

For the animal experimental protocol of IL-6 neutralization, *Tagln*^*OE*^ iMEFs (1.5 × 10^6^) mixed with LLCs cells (5 × 10^5^) were subcutaneously injected into the backs of C57BL/6N mice. One group was injected with an isotype control, and the other group was treated with an IL-6 neutralizing antibody (MP5-20F3; BioXcell). For this treatment, mice were intraperitoneally injected with 100 μg/mouse of IL-6 neutralizing antibody, on days 8, 10 and 12. Mice were treated according to the methods described above. All animal experiments were approved by the Review Board of the First Affiliated Hospital of Zhengzhou University.

### Tumor microarrays (TMAs)

Commercial tissue microarrays (HLugA180Su06, HLugA020PG02) of human lung cancer were obtained from Shanghai Xinchao Biotechnology Co., Ltd. (Shanghai, China), and immunohistochemistry (IHC) was performed as described later (“IHC staining” section). Slides were stained for TAGLN (1:200, Abcam, #ab14106), α-SMA (Abcam, #ab5694), or PDGFR-β (Abcam, #ab32570) and imaged using the Slide Scanner System SQS-1000 (Teksqray). Two fields per slide per patient were double-blinded and quantified for TAGLN staining intensity and percentage (a total of four quantifications were performed per patient and the mean was calculated). For immunohistochemistry scoring, the intensity of staining (0 = negative, 1 = weak, 2 = moderate, 3 = strong) and the percentage of positively stained tumor cells (1 = 0–25%, 2 = 26–50%, 3 = 51–75%, 4 = 75–100%) were used for the quantification. The total IHC score equals the product of the intensity of staining and the percentage of positively stained tumor cells. The total IHC scores ≤6 was defined as low expression, and >6 was defined as high expression [26].

### Cells

#### Human cancer-associated fibroblasts

CAFs and NFs were isolated from lung cancer tissues and benign tissues at least 5 cm from the tumor, respectively, using the outgrowth method described previously [27, 28]. Briefly, sterile fresh surgical tissue was placed on ice in Dulbecco’s modified Eagle medium (DMEM, Hyclone) supplemented with 10× penicillin–streptomycin (1000 U/ml penicillin and 1000 μg/ml streptomycin). The tissue was washed two to three times with 1× phosphate-buffered saline (PBS, Hyclone) to remove blood contamination. The tissue was then cut into fine pieces using a sterile scalpel and digested with DMEM containing type 1A collagenase (Sigma) for 2 h at 37 °C, with agitation every 20 min. Next, the digest was removed, and the debris was washed with DMEM without fetal bovine serum (FBS; PAN-Biotech). The cell suspension was filtered through a 100 μm nylon mesh (BD Biosciences) and centrifuged at 2000 × *g* for 5 min at 4 °C. Cell pellets were then resuspended and cultured in 25 cm^2^ culture flasks (Corning), in DMEM containing 10% FBS supplemented with L-glutamine (2 mmol/l), penicillin, and streptomycin. Cells were cultured at 37 °C in a 5% CO_2_-air humidified atmosphere; CAFs grew out of the tissue blocks 10–14 days later. Human CAFs and NFs were used between the fourth and eighth generation to ensure the maintenance of the phenotypic and functional properties of CAFs and NFs.

#### Mouse cancer-associated fibroblasts

The mCAFs and mNFs were isolated from the lungs of a spontaneous lung cancer mouse model, as previously described [29]. Briefly, mouse lungs were minced and dissociated in DMEM containing 10% FBS, supplemented with L-glutamine (2 mmol/l), penicillin, and streptomycin (herein defined as DMEM medium), with 0.5% of collagenase type I for 1 h at 37 °C in a thermo-shaker. Cell suspensions were centrifuged at 1500 rpm for 5 min, and pellets were resuspended in DMEM medium and plated in culture dishes.

#### Mouse lung cancer cells and embryonic fibroblasts

Mouse LLCs cells were kindly provided by Prof. Yan Li of the Academy of Military Medical Sciences. Immortalized mouse embryonic fibroblasts (iMEFs) were gifted by Prof. Xi Lin of the Tsinghua University. Cells were cultured in high-glucose DMEM supplemented with 10% FBS, 100 U/ml penicillin, and 100 mg/ml streptomycin, incubated at 37 °C in a humidified atmosphere with 5% CO_2_, and tested monthly for detection of mycoplasma contamination. For NF-κB inhibition, cells were pretreated with pyrrolidine dithiocarbamate (PDTC) (25 μM, S3633, Selleck) or SC75741 (8 μM, HY-10496, MedChem Express) for 24 h and then used for subsequent experiments.

#### Lentivirus transfection and selection of stable transfectants

Lentivirus/GV492-Tagln (Ubi-MCS-3FLAG-CBh-gcGFP-IRES-puromycin), lentivirus/GV118-shTagln (U6-MCS-Ubi-EGFP), and the corresponding control lentiviruses were purchased from GeneChem (Shanghai, China). Stable cell lines were constructed using lentiviral gene delivery system. iMEF (1 × 10^5^) were seeded in a six‐well plate and transducer the next day with ∼50% confluency. Cells were transduced with the lentiviruses, following manufacturer’s instructions. To ensure only transduced cells were used, we selected the GFP+ cells through a dual-selection process, using puromycin (presented in the lentiviruses as a resistance cassette) and flow-sorting. Stably infected clones were selected and tested by western blot and qRT-PCR. Multiple stable clones were used to eliminate potential clonal effects. Knockdown clone #1 and clone #2 were selected for subsequent experiments.

### RNA sequencing (RNA-seq) and data analysis

The RNA samples were sent to a commercial gene sequencing company BGI (Shenzhen, China), for library construction and transcriptome sequencing. RNA-seq libraries were prepared using an Illumina RNA-Seq Preparation Kit and sequenced on a HiSeq 2500 sequencer. For RNA-seq data analysis, the Wald test was used to calculate *p* values, with false discovery rate set to a threshold of <0.05. Differentially expressed genes were selected and categorized using Gene Ontology (GO) biological process analysis and Kyoto Encyclopedia of Genes and Genomes (KEGG) pathway enrichment analysis. The data mining and graph presentation process, including Venn diagram, KEGG, heat map, and clustering, were all performed by Dr. Tom, a customized data mining system within the BGI.

### Western blot analysis

Cells were harvested and lysed with RIPA buffer. Protein lysates were quantified using the protein BCA Assay Kit (#23228; Thermo Fisher), according to the manufacturer’s instructions. Equal amounts of protein lysates were resolved by SDS-PAGE, transferred to nitrocellulose membranes (pore size 0.45 µm, Merck Millipore, Darmstadt, Germany), and detected by immunoblotting. The primary antibodies were incubated overnight at 4 °C, followed by immunoblotting with horseradish peroxidase-coupled secondary antibodies for 1 h at 25 °C. The bands corresponding to the interest proteins were visualized using an ECL western blotting Kit (#CW00495; CWBIO) and detected using a ChemiDoc MP Imaging System (Bio-Rad, Hercules, CA, USA). The following primary antibodies were used: TAGLN (#ab14106; Abcam), α-SMA (#ab5694; Abcam), PDGFR-β (#ab32570; Abcam), p-iKKβ (#AP0546; Abclonal), IKKβ (#A0714, Abclonal), p-p65 (#3033S; CST), p65 (#8242S; CST), E-cadherin (#3195S; CST), vimentin (#GTX100619; Genetex), SOX2 (#11064-1-AP; Proteintech), OCT4 (#11263-1-AP; Proteintech), and GAPDH (1:10,000, #AC001; Abclonal). All primary antibodies were used at a dilution of 1:1000, and secondary antibodies (Abclonal, #AS014, #AS003) at 1:3000, unless otherwise stated. Anti-TAGLN antibody was validated by western blot; 293T cells were used as negative controls and mCAFs and Hela cells as positive controls (Supplementary Fig. 1A).

### RNA isolation and quantitative RT-PCR

Total RNA was extracted using TRIzol (#108-95-2; TAKARA) according to standard procedures, and cDNA was synthesized using a reverse transcription kit (#RR036A; TAKARA), following manufacturer’s instructions. Quantitative real-time PCR was performed using SYBR® Green FastMIX® (#RR820A; TAKARA) in a StepOne™ Real-Time PCR System. Primers used in this study are listed in Supplementary Table 1. mRNA expression was calculated using the 2^−ΔΔCt^ method, and GAPDH or 18S RNA was used as a reference for gene expression. The experiments were repeated at least thrice.

### IHC staining

Previously described standard procedures were used for IHC [30, 31]. The primary antibodies used were TAGLN (1:200, #ab14106; Abcam), α-SMA (1:200, #ab5694; Abcam), PDGFR-β (1:200, #ab32570; Abcam,), and IL-6 (1:200, #GB11117; Servicebio). The secondary antibody used for all IHC procedures was the horseradish peroxidase‑conjugated goat anti-rabbit (#CW0103S; CWBIO). For the TAGLN IHC expression analysis, normal mouse bladder tissues with and without primary antibody were used as positive and negative controls, respectively (Supplementary Fig. 1B).

### Immunofluorescence (IF) staining

IF staining was performed according to standard protocols [31, 32]. The primary antibodies used were Ki-67 (1:200, #ab16667; Abcam) and anti-α-SMA (1:200, #ab240654; Abcam). The secondary antibodies used were donkey anti-rabbit Alexa Fluor 488 (1:200, #A21206; Thermo Fisher) and goat anti-mouse Alexa Fluor 555 (1:200, #A21422; Thermo Fisher). Nuclei were stained with 4′,6‐diamidino‐2‐phenylindole (DAPI, Life Technologies) for 5 min. Images were obtained using an inverted fluorescence microscope (Leica). Ki-67 positive cells and Ki-67/α-SMA double-positive cells were counted and averaged for quantitative analysis.

### Time-lapse live cell microscopy imaging

Cell motility was assessed using a confocal microscope (Perkin Elmer Ultra VIEW VOX), according to the manufacturer’s protocol. *Tagln*^*OE*^/*Tagln*^*sh*^ fibroblasts and their corresponding control cells were seeded at a density of 20,000 cells/well in confocal glass-bottom dishes, incubated at 37 °C and 5% CO_2_ for 24 h, to allow for cell attachment. Imaging was then performed for a 2 h period, with images collected every 3 min. Time-lapse images were subsequently analyzed to track and quantify cell motility using ImageJ software (NIH, Bethesda, MD, USA).

### Cell proliferation assay

Cells were seeded into 96-well plates at a density of 2000 cells/well with 100 μl DMEM (with 10% FBS), incubated at 37 °C for different times, as indicated in the figures, followed by incubation with the Cell Counting Kit-8 (CCK-8) solution. Cells in 100 μl of medium were treated with 10 μl of the CCK-8 solution and incubated for 2 h at 37 °C. Absorbance was measured at a wavelength of 450 nm.

### Transwell migration and invasion assay

The cell migration and invasion assay were performed using a proliferation blocker (mitomycin C), to observe the effect of TAGLN on the migratory or invasive potential of cells, without an effect on cell proliferation. The cell migration assay was performed using transwell chambers (8-μm pores, #3422; Corning), while the cell invasion assay was performed using Matrigel-coated (#356234; Corning) transwell chambers (coating on the upper surface). Cells (that migrated or invaded through the Matrigel to the lower surface of the membrane) were fixed with 4% paraformaldehyde for 10 min and stained with 0.5% crystal violet for 30 min, according to standard protocols. Image fields were randomly chosen, and the number of fixed cells was counted using the ImageJ software.

### 3D gel invasion assay

The 3D gel invasion assay was performed as described previously [33, 34]. Briefly, 200 μl of serum-free gel containing Col1a1 (#07001; Stemcell) and Matrigel (#356234; Corning) were used to coat a transwell chamber (3-μm pores, #3415; Corning) in 24-well plates. LLCs were labeled with CellTracker CM-Dil (red) (#40718ES60; Yeasen) following the manufacturer’s instructions, and iMEFs were transfected with green fluorescent protein, as previously described. The cells were mixed (4.5 × 10^4^ cells for each cell type) and placed on the gels in a medium containing 0.2% FBS. DMEM was added to the bottom chamber. After incubation at 37 °C for 7 days, gels were fixed in 4% paraformaldehyde and cut vertically into 50-μm slices using a vibrating microtome (Leica VT-1200S; Leica). Images were obtained using a confocal microscope (LSM880, Zeiss), and the area of invading cells was quantified using the ImageJ software. Invasion index was calculated as follows: invasive index = (invasive cells)/(non-invading cells + invasive cells).

### Conditioned medium stimulation

Transfected iMEFs (2 × 10^6^ cells) were plated into 10 cm^2^ culture dishes, fresh medium (DMEM, 10% FBS, and 1% penicillin–streptomycin) was added the next day, and the cells were grown for the subsequent 3 days. The conditioned medium (CM) was harvested and concentrated at 700 × *g* for 10 min at 4 °C.

### Colony formation assay

For the colony formation assay, 500–1000 cells were seeded in 6-well plates and cultured for 2 weeks. At the indicated time points, cells were fixed with 4% paraformaldehyde, stained with 0.5% crystal violet methanol solution for 30 min and imaged.

### Tumor sphere formation assay

The sphere formation assay was performed in 24-well ultralow-attachment plates (#3473; Corning). LLCs (1000 cells/well) were seeded in serum-free DMEM, containing 10 mM HEPES, 10 ng/ml of basic fibroblast growth factor (#450-33-10 μg; Proteintech,), 2% B27 (serum-free supplement, #17504044; Gibco), and 20 ng/ml of epidermal growth factor (#315-09-100 μg; Proteintech). Each well was examined under a light microscope, and the total number of spheroids was counted.

### Enzyme-linked immunosorbent assay (ELISA)

The levels of interleukin (IL)-6 were quantified using ELISA kits (#KE10007, FineTest), according to the manufacturer’s protocols. Absorbance was measured at 450 nm using a multifunctional microplate reader (Thermo Fisher Scientific). Protein levels were calculated in pg/ml.

### Statistical analyses

Statistical analyses were performed using GraphPad Prism 8 (GraphPad Software Inc.) or IBM SPSS version 23.0. Logistic regression was used for multivariate analysis. Data were tested for Gaussian distribution using the D’Agostino–Pearson omnibus normality test. For Gaussian distributions, a paired or unpaired two-tailed Student’s *t* test was performed for comparisons between two groups, and one-way ANOVA with Tukey’s post test was applied for multiple comparisons. For non-Gaussian distributions, Mann–Whitney and Kruskal–Wallis tests, with Dunn’s post test (for multiple comparisons), were performed. All values are presented as the mean ± standard error of mean (SEM). Differences were considered statistically significant when *p* < 0.05 (*p < 0.05, **p < 0.01, or ***p < 0.001).

## Results

### TAGLN is highly expressed in lung cancer stroma

**Fig. 1:**
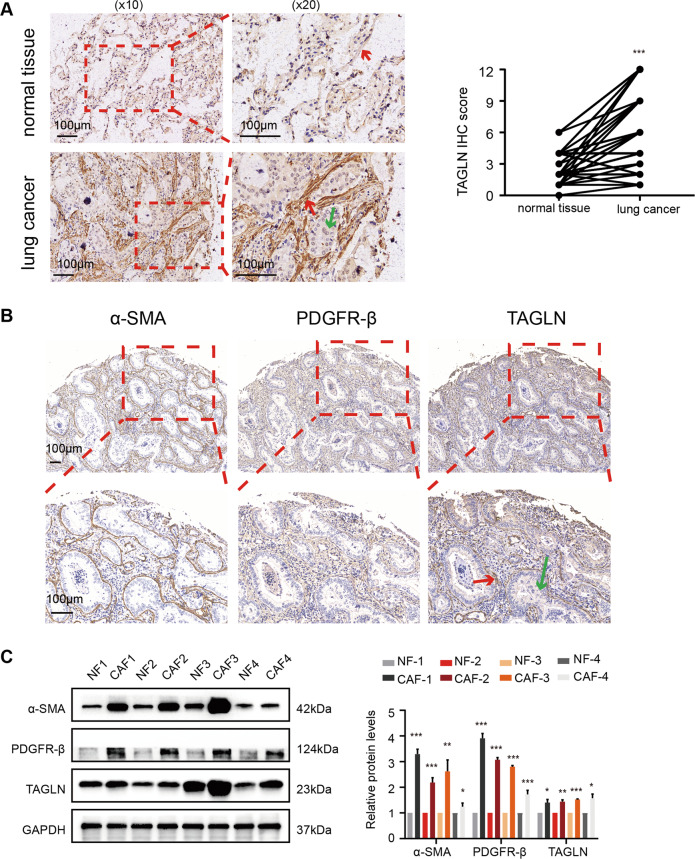
Transgelin is highly expressed in human lung cancer stroma. **A** Immunohistochemical (IHC) staining for transgelin (TAGLN) in lung cancer and adjacent normal tissues from a tissue microarray of 94 tumors and 86 paired adjacent normal tissues. IHC score of transgelin in lung cancer and adjacent tissues. **B** IHC staining for TAGLN, α-SMA, and PDGFR-β in one patient from the human lung cancer tissue microarray (*n* = 10). **C** Western blot analysis of protein levels of α-SMA, PDGFR-β, and TAGLN in CAFs and NFs (replicates from four patients). Red arrows represent stromal fibroblasts, green arrows represent lung cancer cells. Data are represented as mean ± SEM. ***p < 0.001, **p < 0.01, *p < 0.05.

First, we examined TAGLN expression in human lung cancer and adjacent non-tumor tissues through IHC. As shown in Fig. 1A, the expression in the stroma of human lung cancer cells was significantly higher than that in the stroma of normal lung tissue. Co-localization of α-SMA, PDGFR-β, and TAGLN in three sequential sections of human lung cancer tissue microarrays revealed TAGLN localization in stromal fibroblasts (Fig. 1B). To further investigate fibroblasts features and their interaction with tumor cells, CAFs and paired NFs were isolated from human lung cancer and adjacent non-tumor tissues. Fibroblasts were identified as CAFs mainly based on the analysis of α-SMA and PDGFR-β (Fig. 1C and Supplementary Fig. 2A). Western blot analysis confirmed that TAGLN protein levels were markedly higher in CAFs than in NFs (Fig. 1C). Combining these results, we showed that TAGLN exists predominantly in fibroblasts from the tumor stroma. Further data analysis revealed that increased stromal TAGLN expression was correlated with positive lymph node metastasis (*p* = 0.0001), higher Tumor Node Metastasis (TNM) stage (*p* = 0.0003), and higher histopathological grade (*p* = 0.0383) (Table 1). Multivariate logistic regression analysis revealed that the size of tumor was a significant independent prognostic factor (odds ratio (OR) = 6.2532, *p* = 0.0178) (Table 2). Moreover, stromal TAGLN expression was higher in metastatic tissues than in nonmetastatic cancer tissues (Supplementary Fig. 2B). Based on these findings, we surmise that stroma-derived TAGLN may be associated with human lung cancer metastasis.

**Table 1:** Clinicopathological variables of patient samples and expression of transgelin in lung cancer stroma.

Variables	Patients	TAGLN low (%)	TAGLN high (%)	*χ*^2^	*p* value
Age (years)					
≥60	51	18 (35.29%)	33 (64.71%)	3.140	0.0764
<60	43	23 (53.49%)	20 (46.51%)		
Gender					
Male	53	25 (47.17%)	28 (52.83%)	0.6237	0.4297
Female	41	16 (39.02%)	25 (60.98%)		
Size of tumor					
<5 cm	70	37 (52.86%)	33 (47.14%)	9.518	0.0020
≥5 cm	24	4 (16.67%)	20 (83.33%)		
Lymph node status					
Negative	43	28 (65.12%)	15 (34.88%)	14.90	0.0001
Positive	51	13 (25.49%)	38 (74.51%)		
T-primary tumor					
T1 + T2	70	38 (54.29%)	32 (45.71%)	12.69	0.0004
T3 + T4	24	3 (12.5%)	21 (87.5%)		
TNM stages					
I–II	51	31 (60.78%)	20 (39.22%)	13.36	0.0003
III–IV	43	10 (23.26%)	33 (76.74%)		
Pathological grade					
I	11	8 (72.73%)	3 (27.27%)	4.293	0.0383
II–III	83	33 (39.76%)	50 (60.24%)		

Chi-square tests for all analyses.

*TAGLN* transgelin.

**Table 2:** Univariate and multivariate analysis of factors associated with transgelin expression.

	Univariate analysis			Multivariate analysis		
Variables	OR	95% CI	*p* value	OR	95% CI	*p* value
Age (<60 years vs. ≥60 years)	0.4743	0.2055–1.058	0.0764	1.9132	0.6406–5.7142	0.2452
Gender (male vs. female)	1.395	0.6335–3.196	0.4297	1.7803	0.5914–5.3595	0.3050
Size of tumor (<5 cm vs. ≥5 cm)	5.606	1.784–16.15	0.0020	6.2532	1.3738–28.4632	0.0178
Lymph node status (Negative vs. Positive)	5.456	2.290–13.26	0.0001	0.2628	0.0653–1.0572	0.0599
T-primary tumor (T1 + T2 vs. T3 + T4)	8.313	2.300–27.79	0.0004	0.2402	0.0507–1.1375	0.0722
TNM stages (I–II vs. III–IV)	5.115	2.113–12.18	0.0003	1.7055	0.4182–6.9552	0.4567
Pathological grade (I vs. II–III)	4.040	1.084–14.69	0.0383	0.1439	0.0072–2.8735	0.2044

*OR* odds ratio, *CI* confidence interval.

**Fig. 2:**
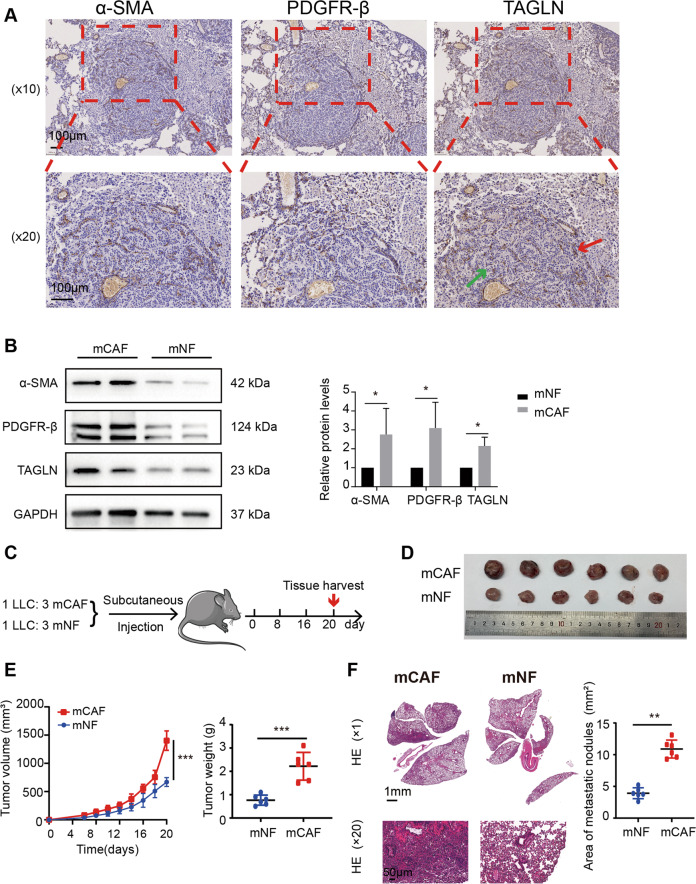
CAFs facilitate tumor growth and lung metastasis in vivo. **A** IHC staining for TAGLN, α-SMA, and PDGFR-β in the lung cancer tissue from EGFR^L858R^ transgenic mice (*n* = 6). **B** Western blot analysis of protein levels of α-SMA, PDGFR-β, and TAGLN in mCAFs and mNFs (*n* = 3). **C** Diagram of the mouse model with subcutaneous tumor implantation. **D** Photographs of tumors from mice. **E** Tumor volume and tumor weight. **F** Representative hematoxylin and eosin staining images and quantification data of lung metastasis in mice. Upper panel scale bar: 1 mm; lower panel scale bar: 50 µm. Results are shown as mean ± SEM and compared by unpaired *t*-test. ***p < 0.001, **p < 0.01, *p < 0.05.

Furthermore, TAGLN was mainly expressed in the stromal region in the EGFR-driven spontaneous lung cancer mice (Fig. 2A). Additionally, TAGLN expression levels were markedly higher in mCAFs than in mNFs (Fig. 2B). In vivo experiments showed that mCAFs significantly promoted tumor growth and metastasis, compared with mNFs (Fig. 2C–F). Taken together, these data show not only that TAGLN is highly expressed in lung CAFs but also that this high expression may be associated with the metastasis process.

### TAGLN promotes fibroblasts activation

**Fig. 3:**
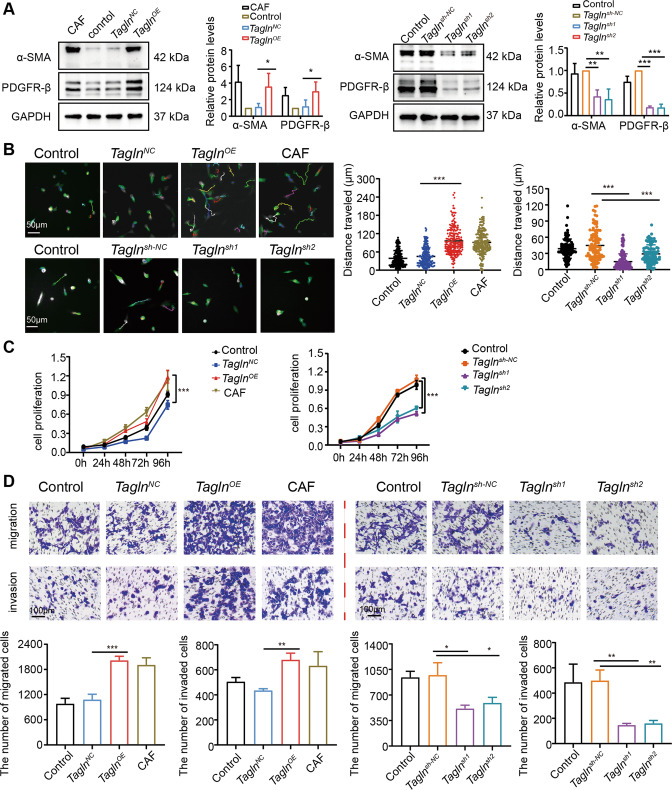
Transgelin promotes fibroblasts activation. **A** The effect of transgelin (TAGLN) on the protein levels of cancer-associated fibroblast (CAF) markers, evaluated by western blot (*n* = 3). **B** Representative images of time-lapse microscopy imaging of immortalized mouse embryonic fibroblast (iMEF) overexpressing *Tagln* or with *Tagln* knockdown. Each color line represents the tracking of a different cell. Total distance traveled for different fibroblasts. (*Tagln*^*OE*^ iMEF *n* = 240, *Tagln*^*sh*^ iMEF *n* = 120). Scale bar: 50 µm. **C** Changes in iMEF cell proliferation with *Tagln* overexpression or knockdown. **D** Effects of *Tagln* overexpression or knockdown on cell migration and invasion. ***p < 0.001, **p < 0.01, *p < 0.05.

Considering the stability and repeatability of the experimental results, we used an iMEF cell line to construct an in vitro experimental system. We first investigated whether TAGLN affects the phenotypic conversion of fibroblasts. We established *Tagln*^*OE*^ and *Tagln*^*sh*^ iMEF cell lines by infection with the corresponding lentiviruses or subsequent experiments. The knockdown of *Tagln* (*Tagln*^*sh*^) was confirmed in two separate clones of iMEFs, *Tagln*^*sh1*^ and *Tagln*^*sh2*^. Successful overexpression and knockdown were confirmed by qRT-PCR and western blot analysis (Supplementary Fig. 3A). We found higher protein and mRNA levels of α-SMA and PDGFR-β, which are CAF markers, in *Tagln*^*OE*^ cells, compared to negative control-transfected cells (Fig. 3A and Supplementary Fig. 3B). Contrarily, mRNA and protein levels of α-SMA and PDGFR-β were markedly reduced in *Tagln*^*sh*^ fibroblasts (Fig. 3A and Supplementary Fig. 3B). Time-lapse microscopy revealed that *Tagln* overexpression significantly increased iMEFs mobility (Fig. 3B) and proliferation (Fig. 3C). Using a transwell assay, we observed that *Tagln* overexpression remarkably promoted iMEFs migration and invasion (Fig. 3D). *Tagln*^*sh*^ fibroblasts exhibited decreased motility, proliferation, and migration/invasion (Fig. 3B–D). Taken together, these findings suggest that TAGLN activates fibroblasts and, in turn, induces their pro-tumor phenotype.

### Tagln-overexpressing fibroblasts promote cell migration, invasion and cancer cell stemness in lung cancer cells

**Fig. 4:**
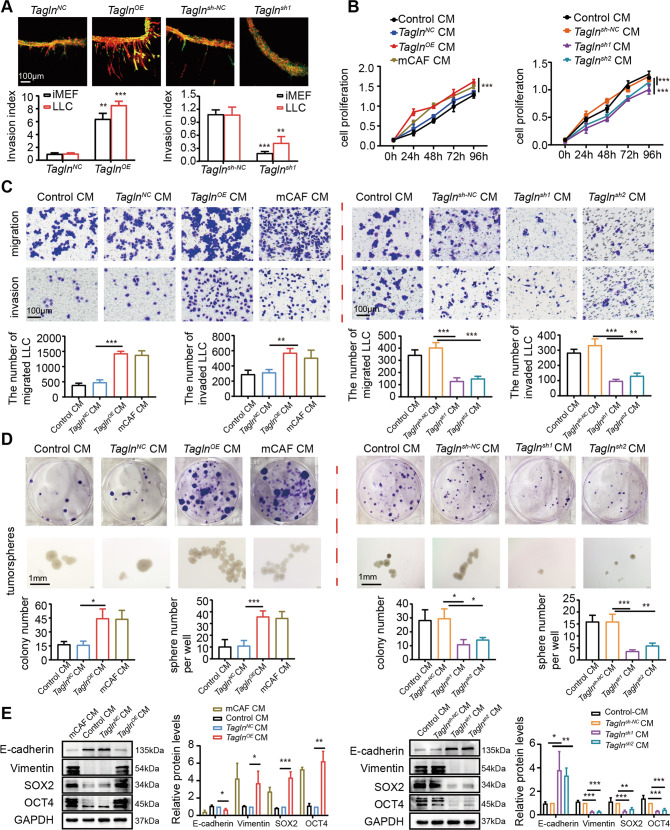
*Tagln*^*OE*^ fibroblasts promote cell migration, invasion, and cancer cell stemness in lung cancer cells. **A** Immortalized mouse embryonic fibroblasts (iMEFs; green) and Lewis lung cancer cells (LLCs; red) were subjected to a 3D gel invasion assay. Scale bar: 100 µm. **B** Effect of *Tagln* overexpression or knockdown on LLCs proliferation. **C** Effects of conditioned medium (CM) derived from *Tagln*^*OE*^ iMEFs or *Tagln*^*sh*^ iMEFs on LLC migration and invasion. Scale bar: 100 µm. **D** Colony formation by LLCs with CM from T *Tagln*^*OE*^ iMEFs or *Tagln*^*sh*^ iMEFs. The sphere numbers of LLCs were counted by ImageJ software. Scale bar: 1 mm. **E** Western blot analysis of epithelial–mesenchymal transition-related proteins and cancer stem cell markers in LLCs with different CM. Data are represented as mean ± SEM from at least three independent experiments. ***p < 0.001; **p < 0.01; *p < 0.05.

CAFs are known to contribute to tumor development and progression by regulating the malignant phenotype of tumor cells [35–37]. To investigate the function of *Tagln*-overexpressing fibroblasts in tumor cells, we performed 3D-gel invasion assays. Results showed increased LLCs cell invasion (Fig. 4A) after indirect co-culture with *Tagln*^*OE*^ iMEFs. Next, we cultured LLCs with CM derived from *Tagln*^*OE*^ iMEFs (and control cells), and observed increased LLCs proliferation (Fig. 4B). Furthermore, a transwell assay was used to detect the migration and invasion abilities of LLCs. As shown in Fig. 4C, *Tagln*^*OE*^ iMEFs-derived CM increased LLCs migration and invasion. Additionally, the number of colonies formed as well as LLCs number was also significantly elevated (Fig. 4D). Western blot results showed higher protein levels of cancer stem cell markers, including SOX-2 and OCT-4, in cancer cells cultured in *Tagln*^*OE*^ iMEFs-derived CM, compared to those in the negative control groups (Fig. 4E). E-cadherin was downregulated, and vimentin was upregulated in the CM-treated *Tagln*^*OE*^ iMEF group, indicating higher activation of the EMT program (Fig. 4E). Additionally, OCT-4, SOX-2, E-cadherin, and vimentin mRNA levels showed similar trends as protein levels (Supplementary Fig. 4).

We next tested whether silencing *Tagln* in iMEFs affected the tumor-supportive function. In 3D-gel invasion assays, the mixture of *Tagln*^*sh*^ iMEFs and LLCs did not show effective invasive properties. *Tagln* knockdown in iMEFs inhibited cancer cell invasion (Fig. 4A) and decreased proliferation of LLCs (Fig. 4B). Additionally, *Tagln* knockdown in iMEFs effectively inhibited the migration and invasion of LLCs (Fig. 4C), as well as colony formation capacity and tumorigenesis (Fig. 4D). Moreover, CM from *Tagln*^*sh*^ iMEFs decreased SOX-2 and OCT-4, increased E-cadherin, and downregulated vimentin expression in LLCs (Fig. 4E). Together, these results indicate that *Tagln* overexpression in fibroblasts might enhance the malignant phenotype of LLCs.

### Tagln-overexpressing fibroblasts promote the growth and spread of lung cancers

**Fig. 5:**
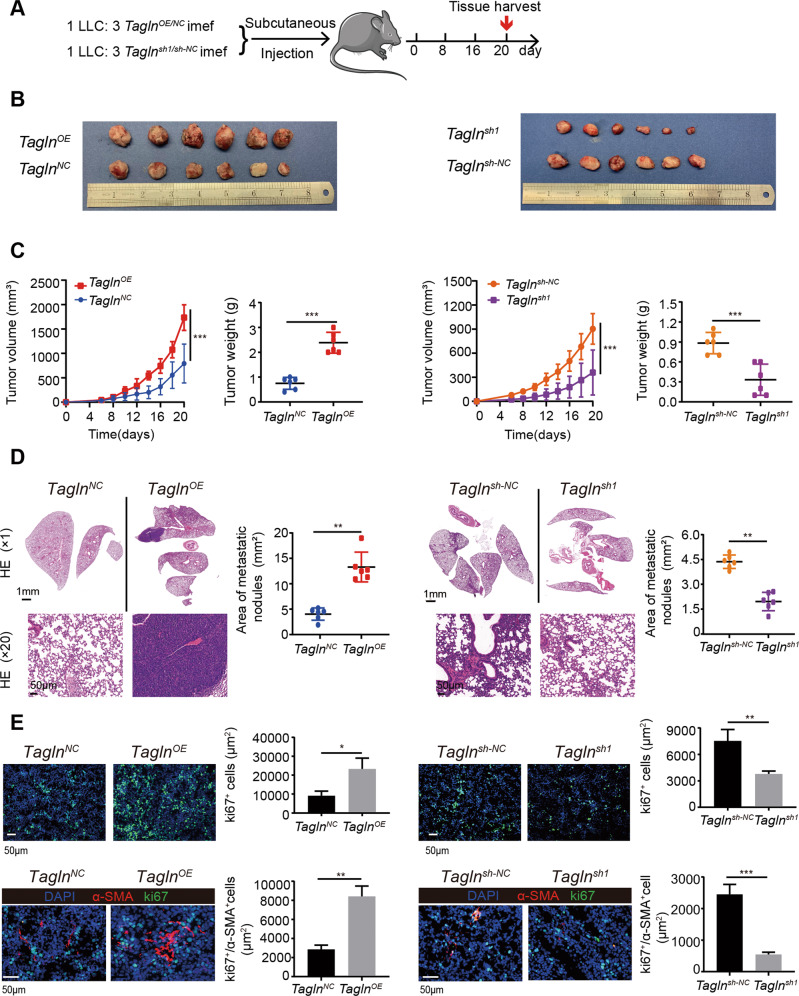
*Tagln*^*OE*^ fibroblasts promote the growth and spread of lung cancers. **A** Scheme of the mouse experiment design. **B** Images of the tumors from the different groups. **C** Tumor growth curves and weights (*n* = 6); ***p < 0.001. **D** Representative hematoxylin and eosin staining images of lungs and areas of lung metastasis for each group. Upper panel scale bar: 1 mm; lower panel scale bar: 50 µm. Results are represented as mean ± SEM and compared by unpaired *t*-test. **p < 0.01. **E** Immunofluorescence staining for Ki-67 (green) and nuclear staining DAPI (blue) of tumors from the different groups (upper panels). Immunofluorescence staining for α-SMA (red), Ki-67 (green), and nuclear staining DAPI (blue) of tumors from the different groups (lower panels). Quantification of co-expression of α-SMA^+^ and Ki-67^+^ cells. Data are represented as mean ± SEM from at least three independent experiments.

To further explore the importance of transgelin in tumor progression in vivo, we mixed LLCs and *Tagln*^*OE*^ iMEFs or *Tagln*^*sh1*^ iMEFs, at a ratio of 1:3 and inoculated them into C57BL/6 mice to establish a model of subcutaneous tumor transplantation (Fig. 5A). As shown in Fig. 5B, C, the tumor volume and weight in the *Tagln*^*OE*^ iMEFs group were significantly increased compared with those in the control group. Moreover, the co-injection of *Tagln*^*OE*^ iMEFs and LLCs promoted LLC metastasis, as shown in the HE staining images (Fig. 5D).

Ki-67 is a well-known cell proliferation marker that correlates with tumor aggressiveness and is considered a prognostic parameter. We found higher Ki-67 levels in tumor tissues of the *Tagln*^*OE*^ iMEF group than in the control group. Furthermore, the number of α-SMA^+^ and α-SMA^+^/Ki-67^+^ cells also increased in the *Tagln*^*OE*^ iMEF group (Fig. 5E). Results from mice with subcutaneous tumor transplantation with the *Tagln*^*sh*^ iMEFs further supported these observations (Fig. 5B–E). Taken together, the in vivo data supports the hypothesis that *Tagln*-overexpressing fibroblasts may promote tumor growth and spreading.

### Tagln-overexpressing fibroblasts release more IL-6 via the activation of the NF-κB signaling pathway

**Fig. 6:**
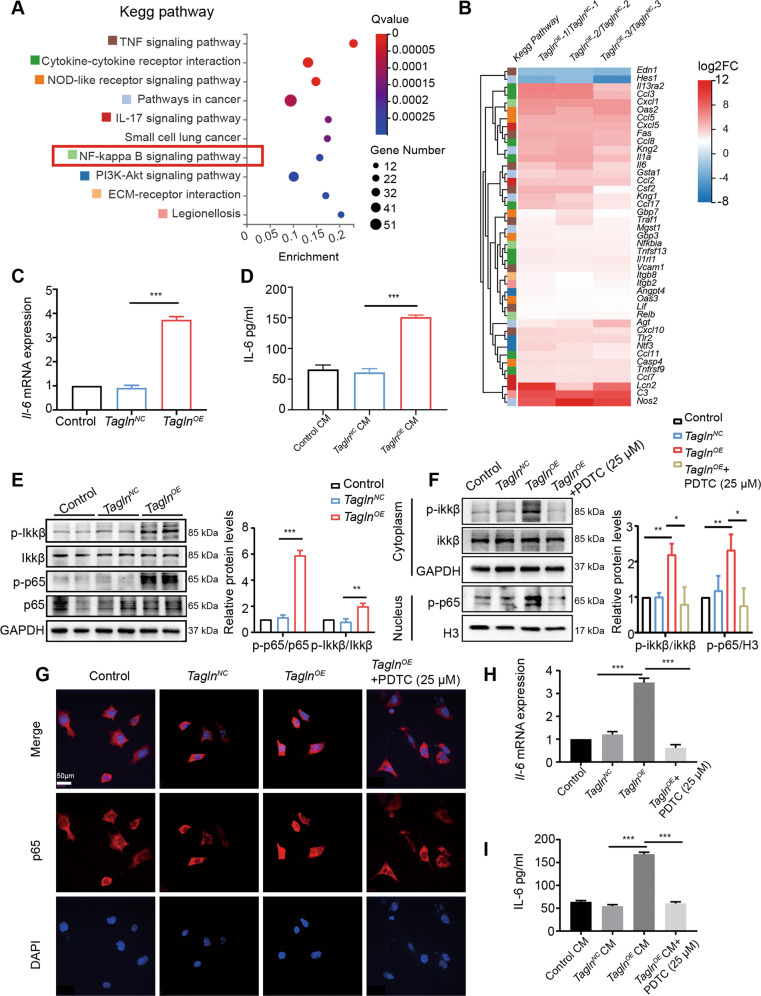
*Tagln*^*OE*^ fibroblasts release more IL-6 via the activation of the NF-κB signaling pathway. **A** Bubble map representing significantly enriched pathways according to the Kyoto Encyclopedia of Genes and Genomes (KEGG) pathway analysis conducted for differentially expressed genes. **B** Heatmap representing the log_2_ fold changes of differentially expressed genes between *Tagln*^*OE*^ iMEFs and *Tagln*^*NC*^ iMEFs involved in the major pathways identified through KEGG analysis. **C** *Tagln* overexpression upregulated the mRNA levels of cytokines *Il-6* in iMEFs. **D** ELISA analysis confirmed the increased protein levels of secreted IL-6 in the conditioned medium from *Tagln*^*OE*^ iMEFs. **E** Western blot analysis of proteins of the NF-κB signaling pathway, activated by *Tagln* overexpression. **F** Increased protein levels of p-p65 in the nucleus of iMEFs, prevented by NF-κB inhibitor pyrrolidine dithiocarbamate (PDTC). **G** p65 translocation to the nucleus assessed through immunofluorescence assay. **H** Quantitative RT-PCR revealed that PDTC obviously inhibited the upregulated *Il-6* mRNA levels caused by transgelin overexpression in iMEFs. **I** ELISA analysis confirmed that PDTC markedly prevented the increased levels of secreted IL-6 in the conditioned medium from *Tagln*^*OE*^ iMEFs. Data are represented as mean ± SEM from at least three independent experiments. ***p < 0.001; **p < 0.01; *p < 0.05.

RNA-seq and bioinformatic analyses were performed to analyze differentially expressed genes in *Tagln*^*OE*^ iMEFs. In total, 725 gene were upregulated and 273 were downregulated (Supplementary Fig. 5A, *p* < 0.05). KEGG pathway analysis was performed on the 998 differentially expressed genes. A bubble map showed that these 998 genes were enriched in the TNF signaling pathway, cytokine-cytokine receptor interaction, and NF-κB signaling pathway (Fig. 6A). The differences in gene expression of in these major pathways between *Tagln*^*OE*^ iMEFs and control iMEFs are illustrated as a heatmap (Fig. 6B). Details of the genes enriched in the 10 KEGG pathways are listed in Supplementary Table 2. Through qRT-PCR we verified that *Il-6* was upregulated in *Tagln*^*OE*^ iMEFs (Fig. 6C). Moreover, we observed increased IL-6 secretion in the culture medium supernatants of *Tagln*^*OE*^ iMEFs (Fig. 6D). We found that *Tagln*^*OE*^ iMEFs exhibited enhanced p-IKKβ and p-p65 expression, associated with the activation of the NF-κB signaling pathway (Fig. 6E). In contrast, *Tagln*^*sh*^ iMEFs exhibited decreased p-IKKβ and p-p65 expressions (Supplementary Fig. 5B). These data were consistent with the RNA-seq results. To further confirm the activation of the NF-κB signaling pathway, *Tagln*^*OE*^ iMEFs were treated with PDTC, a potent NF-κB inhibitor that prevents IκB phosphorylation and blocks NF-κB translocation to the nucleus, thereby reducing the expression of downstream cytokines [38, 39]. PDTC prevented the *Tagln*-induced increase in cytoplasmic p-IKKβ and nuclear p-p65 protein levels (Fig. 6F). IF staining further confirmed that *Tagln* overexpression facilitated p-p65 translocation to the nucleus (Fig. 6G). Moreover, PDTC inhibited *Tagln*-induced IL-6 secretion and mRNA expression (Fig. 6H, I). Another NF-κB inhibitor SC75741, had an effect that was close to that of PDTC (Supplementary Fig. 5C–E). These results suggest that *Tagln*-overexpressing fibroblasts promote the release of inflammatory cytokines via the activation of the NF-κB signaling pathway.

### IL-6 from Tagln-overexpressing fibroblasts may promote the malignant phenotype of lung cancer cells

**Fig. 7:**
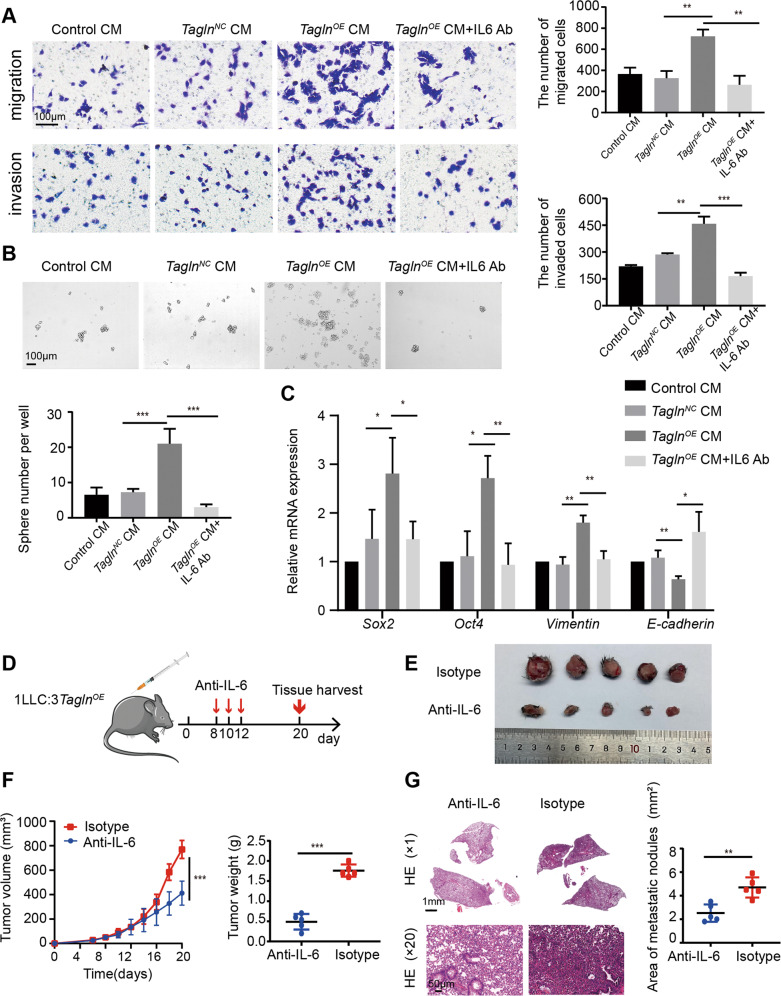
IL-6 from *Tagln*^*OE*^ iMEFs may promote the malignant phenotype of lung cancer cells. **A** Transwell assay was performed to determine the effects of IL-6 neutralizing antibody on migration and invasion properties of Lewis lung cancer cells (LLCs) fed with different conditioned media (CM). **B** Tumor sphere formation assay upon IL-6 neutralizing antibody treatment. **C** Quantitative RT-PCR analysis of mRNA levels of epithelial–mesenchymal transition-related proteins and cancer stem cell markers in LLCs fed with different CM, with or without IL-6 neutralizing antibodies. **D** Anti-IL-6-neutralizing antibody treatment in mice. **E** Photographs of tumors from mice. **F** Tumor volume and tumor weight (*n* = 5). **G** Representative hematoxylin and eosin staining images and quantification data of lung metastasis in mice. Upper panel scale bar: 1 mm; lower panel scale bar: 50 µm. Data are represented as mean ± SEM from at least three independent experiments. ***p < 0.001; **p < 0.01; *p < 0.05.

IL-6 is associated with tumor growth, differentiation, apoptosis, drug resistance, and immune regulation. Furthermore, IL-6 levels have been reported as significantly increased in patients with advanced tumors [40, 41]. Moreover, IL-6 is considered important for lung cancer development [42]. To further investigate whether *Tagln*-overexpressing fibroblasts promoted the malignant phenotypes of LLCs via IL-6, we blocked IL-6 secreted by *Tagln*^*OE*^ iMEFs. With a transwell assay we showed that IL-6 neutralizing antibodies could prevent the invasion and migration of LLCs cultured with CM from *Tagln*^*OE*^ iMEFs (Fig. 7A), and suppress the number of tumor spheres in LLCs (Fig. 7B). Additionally, IL-6 neutralizing antibodies also reversed the expression profile of cancer stem cells and EMT markers induced by CM from *Tagln*^*OE*^ iMEFs (Fig. 7C). These results suggest that IL-6 secreted from *Tagln*^*OE*^ iMEFs may promote the malignant phenotype of LLCs. To further support these findings, we randomly selected subcutaneously transplanted tumor-bearing mice for IL-6 neutralization treatment (Fig. 7D). Treatment with anti-IL6 promoted a decreasing trend in tumor volume and weight (Fig. 7E, F). In addition, IL-6 neutralization also improved lung metastasis (Fig. 7G).

## Discussion

**Fig. 8:**
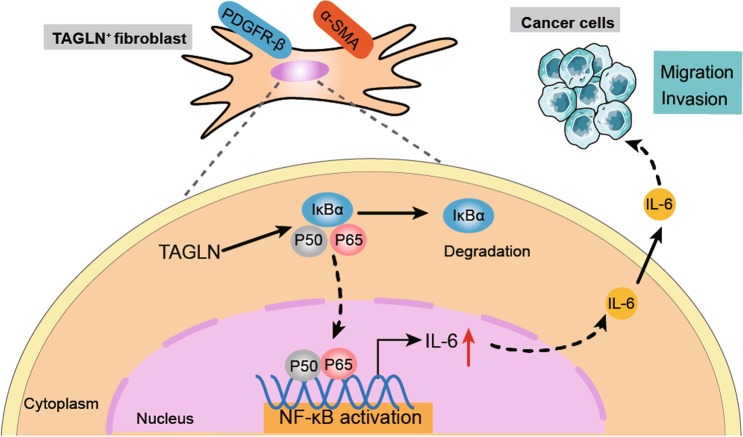
TAGLN promotes lung cancer progression via activation of cancer-associated fibroblasts with enhanced IL-6 release. TAGLN promoted the pro-tumor phenotype of fibroblasts and increased their secretion of pro-inflammatory cytokines, such as IL-6, via the activation of the NF-κB signaling pathway which in turn regulates lung cancer cells migration/invasion.

In the current study, we found that high fibroblastic TAGLN expression in human lung cancer is associated with increased cancer cell lymph node metastasis. Fibroblasts overexpressing *Tagln* promoted the malignant phenotype of LLCs, and the *Tagln*-induced fibroblast activation facilitated the release of inflammatory cytokines via the activation of the NF-κB signaling pathway. IL-6 secreted by TAGLN-positive fibroblasts may promote lung cancer progression (Fig. 8). Together, these findings suggest that TAGLN expression is essential for fibroblasts to acquire the CAF phenotype, which plays an important role in lung cancer progression.

TAGLN, a member of the calmodulin family, acts as an actin-binding protein and regulates cytoskeletal remodeling, through the promotion of actin aggregation [12]. Previous studies have highlighted TAGLN as a tumor metastasis initiator [21]. Increased TAGLN levels have also been associated with prognosis and metastasis in certain tumors, such as esophageal [43], pancreatic [17], and colorectal [14]. There are only few studies on the role of TAGLN in lung cancer, especially since there are no reports on the function of TAGLN in the lung cancer stroma.

In this study, we showed a correlation between cancer cell lymph node metastasis and high stromal TAGLN levels using TMAs. Enhanced stromal TAGLN levels are considered an independent risk factor for lymph node metastasis. Our data are in line with those of a previous study in gastric cancer, in which TAGLN overexpression in stromal fibroblasts promoted tumor metastasis [22]. Although we did not directly observe tumor metastasis in vivo, the pathological morphology of subcutaneously transplanted tumors suggests that *Tagln*^*OE*^ iMEFs tend to promote metastasis. High TAGLN levels were negatively associated with survival and disease-free survival in colon adenocarcinoma, whereas low levels were positively correlated with survival in patients with stage III colorectal cancer [14, 44]. However, in the present study, we did not observe any correlation between overall survival and stromal TAGLN levels. A larger sample size and more detailed clinical staging information are required to further characterize the effects of CAF-derived TAGLN on lung cancer survival.

Recently, Zhou et al. identified five genes (*BGN*, *RCN3*, *TAGLN*, *MYL9*, and *TPM2*) as fibroblast-specific markers for prediction of poor prognosis in colorectal cancer [24]. Similarly, TAGLN was reported as a specific marker of CAFs in the mesenchymal stroma of pancreatic ductal adenocarcinoma [45]. In line with these studies, we demonstrated that *Tagln* overexpression activates normal fibroblasts and promotes the shift to the CAF phenotype in vitro. However, understanding the specific mechanism of fibroblast activation by *Tagln* overexpression requires further studies.

It is well-known that CAFs are a heterogeneous population in TME [46]. As one of the markers of CAFs, *Tagln*-positive fibroblasts may represent an aspect of CAF heterogeneity. Lately, Zheng’s team identified seven CAF subtypes through high-resolution clustering of the integrated data, termed pan-CAF 1-7 [47]. These pan-CAF subtypes were present in the three cancer types (including lung cancer). The results showed that pan-CAF 1 was classified as pan-myCAFs based on elevated expression of activated fibroblast markers (*ACTA2*) and smooth muscle cell markers (*MYH11*, *MCAM*, *Tagln*, and *MYLK*). In a recent paper published by our group, we used single-cell sequencing to group CAF cells from mice that were subcutaneously inoculated with LLC transplant tumors, resulting in the identification of 11 distinct CAF clusters [48]. Among them, cluster 10 exhibited high expression levels of both *ACTA2* and *Tagln* (unpublished data). The above studies suggest that this particular group of CAFs with high expression of *Tagln* may be associated with the myofibroblast-associated CAFs (myCAFs) subtype. Moreover, in the present study, we observed a significant increase in the expression of α-SMA in iMEF cells following *Tagln* overexpression, while the opposite was observed after *Tagln* knockdown. The same trend was seen in subcutaneously transplanted tumors in mice. These results imply that perturbing *Tagln* expression may have an impact on α-SMA-positive myCAFs. However, the exact mechanism remains to be investigated. In addition, future studies should complement the α-SMA/TAGLN double-staining assay in human lung cancer tissues, and subject the staining results to multivariate analysis in relation to clinical variables to further correct for tumor cellularity.

In the current study, we observed that fibroblasts overexpressing *Tagln* were able to promote the malignant phenotype of lung cancer cells, including invasion and migration abilities, EMT, and cancer cell stemness. Furthermore, we explored the possible molecular biological mechanisms involved in these *Tagln*-mediated effects. Our RNA-seq data showed that *Tagln* overexpression can alter several inflammatory pathways, including the NF-κB and TNF signaling pathways, indicating that *Tagln* may participate in the crosstalk between cancer cells and CAFs by mediating the inflammatory process in the TME. Consistent with this, our in vitro results showed that *Tagln*^*OE*^ iMEFs were able to promote p-p65 translocation into the nucleus, which in turn activates the NF-κB signaling pathway and leads to IL-6 production. TAGLN can bind to the Poly (ADP-ribose) polymerase-1 (PARP1) promoter [49], and the PAR-dependent formation of a nuclear PARP1-IKKγ signalosome can promote IKK activation [50]. This may be one of the mechanisms through which TAGLN activates the NF-κB signaling pathway. We will further analyze this mechanism in a follow-up study. CAFs secrete different cytokines that promote tumor progression [51, 52]. IL-6 is one of these, a multifunctional molecule involved in regulating immune and inflammatory responses, and known to promote tumor growth and cancer cells invasion [53, 54]. Blocking IL-6/STAT3 signal transduction can significantly inhibit tumor growth and STAT3 phosphorylation in mice xenografts with non-small cell lung cancer [55]. Our in vivo studies identified an immune suppressive environment upon *Tagln* overexpression, consistent with those described above (Supplementary Fig. 6). Moreover, pro-inflammatory cytokines enhance CAF glycolysis [56], which may result in local energy-rich metabolites and tumor growth. This may occur due to the CAFs transgelin-induced secretion of pro-inflammatory cytokines, ultimately accelerating tumor progression. However, this hypothesis also requires confirmation in future studies. Noteworthily, recent studies have provided insight into the regulation of TAGLN by transforming growth factor (TGF) -beta. Chen et al. found that TGF-β-mediated migration was abolished by TAGLN suppression in bladder cancer [13]. Yu et al. identified *Tagln* as a target of the TGF-β/Smad3-dependent gene expression in alveolar epithelial type II (ATII) cells [57]. However, whether TGF-β can also act as an upstream regulator gene of TAGLN in fibroblasts, thus inducing a positive feedback loop, is currently unknown and requires further studies.

Nevertheless, we acknowledge the limitations of this study. RNA-seq and subsequent experimental analyses demonstrated that high *Tagln* expression in iMEFs promoted the pro-tumor phenotype of fibroblasts and increased IL-6 secretion via the activation of the NF-κB signaling pathway, by enhancing the phosphorylation of IKKβ and p65. However, TAGLN has no phosphokinase activity, therefore the associated mechanisms of NF-κB activation require further exploration. Combining *Tagln* knockdown with TAGLN mutants might help to detect phenotypic changes that could provide some mechanistic insights. Secondly, in the present study, we focused on IL-6 as it is a key inflammatory cytokine involved in different types of cancer [58–62]. However, it remains unexplored whether the same observations would occur for other cytokines. Additionally, considering that one of the key characters of metastatic cells is chemoresistance, future works should focus on the role of TAGLN in chemoresistance. Thirdly, we did not assess the correlation between TAGLN stromal expression and potential mutations of frequent oncogenes in lung cancer. Therefore, we were unable to determine whether tumor cells contribute to high TAGLN levels which in turn promotes proliferation of lung cancer cells and metastasis.

## Conclusion

Here we identified stromal TAGLN as a predictive factor for lymph node metastasis in human lung cancer. We showed that *Tagln* overexpression in fibroblasts promotes lung cancer cell migration and invasion, which may be related to IL-6 secretion resulting from the increased activation of the NF-κB signaling pathway. These findings provide a substructure for further understanding the mechanism by which stromal TAGLN regulates inflammation in the TME, suggesting CAF-derived TAGLN as a potential target for lung cancer therapy.

## Supplementary information

Supplementary figure legends

supplementary figure 1

supplementary figure 2

supplementary figure 3

supplementary figure 4

supplementary figure 5

supplementary figure 6

Supplementary Table 1

Supplementary Table 2

## Data Availability

All RNA-Seq data are available through the NCBI Sequence Read Archive under the accession number PRJNA933602. The raw reads of our transcriptome data have been deposited into the Sequence Read Archive (SRA) database under accession number SRP421859. The datasets generated during and/or analyzed during the current study are available from the corresponding author on reasonable request.
