# Supplementary material for: Transgelin promotes lung cancer progression via activation of cancer-associated fibroblasts with enhanced IL-6 release
Source: Oncogenesis. 2023 Mar 29;12(1):18. doi: 10.1038/s41389-023-00463-5 (PMC10060230; doi:10.1038/s41389-023-00463-5)
Supplement: Supplementary file 1 — Supplementary figure legends [file 41389_2023_463_MOESM1_ESM.docx]

**Supplementary Figure legends**

**Supplementary Figure 1.**

Anti-transgelin (TAGLN) antibody validation through western blot (A) and immunohistochemistry (IHC) staining (B).

**Supplementary Figure 2.**

1. Morphological images of human normal fibroblasts (NFs) and cancer-associated fibroblasts (CAFs). Scale bar: 100 µm. (B) IHC staining for TAGLN in non-lymph node metastatic tumors and lymph node metastatic tumors. Red arrows represent stromal fibroblasts, green arrows represent lung cancer cells. Scale bar: 100 µm.

**Supplementary Figure 3.**

Western blot of immortalized mouse embryonic fibroblasts (iMEFs) infected with a lentivirus-mediated *Tagln*-overexpressing vector or *Tagln* shRNAs. Quantitative RT-PCR confirmation of *Tagln* overexpression or knockdown in iMEFs. (B) Quantitative RT-PCR analysis of the effect of *Tagln* overexpression or knockdown on the mRNA expression of CAF markers. Data are represented as mean ± SEM from at least three independent experiments. ****P* < 0.001; ***P* < 0.01; **P* < 0.05.

**Supplementary Figure 4.**

Quantitative RT-PCR analysis of mRNA levels of epithelial–mesenchymal transition-related proteins and cancer stem cell markers in mouse Lewis lung cancer cells (LLCs) fed with conditioned media from different iMEFs. Data are represented as mean ± SEM from at least three independent experiments. ****P* < 0.001; ***P* < 0.01; **P* < 0.05.

**Supplementary Figure 5.**

(A)Upregulated and downregulated genes in *Tagln^OE^*/*Tagln^NC^* iMEFs (fold change ≥ 1.0, *P* < 0.05). (B) Western blot analysis of proteins of the NF-κB signaling pathway in *Tagln^sh^* iMEFs. (C) Western blot analysis of proteins of the NF-κB signaling pathway, when *Tagln^OE^* iMEFs treated with SC75741. Data are represented as mean ± SEM from at least three independent experiments. ****P* < 0.001; ***P* < 0.01; **P* < 0.05. (D) Quantitative RT-PCR analysis of *Il-6* mRNA levels of *Tagln^OE^* iMEFs treated with SC75741. (E) ELISA was performed to detect the IL-6 secretion of *Tagln^OE^* iMEFs after treatment with SC75741.

**Supplementary Figure 6.**

IHC staining identifying the immune suppressive environment (IL-6 upregulation) upon *Tagln* overexpression.

**Supplementary Table 1.**

List of primers sequences

**Supplementary Table 2.**

Detailed information of the enriched genes in the 10 KEGG pathways.
