# Supplementary figures and images for: Transgelin promotes lung cancer progression via activation of cancer-associated fibroblasts with enhanced IL-6 release
Source: Oncogenesis. 2023 Mar 29;12(1):18. doi: 10.1038/s41389-023-00463-5 (PMC10060230; doi:10.1038/s41389-023-00463-5)

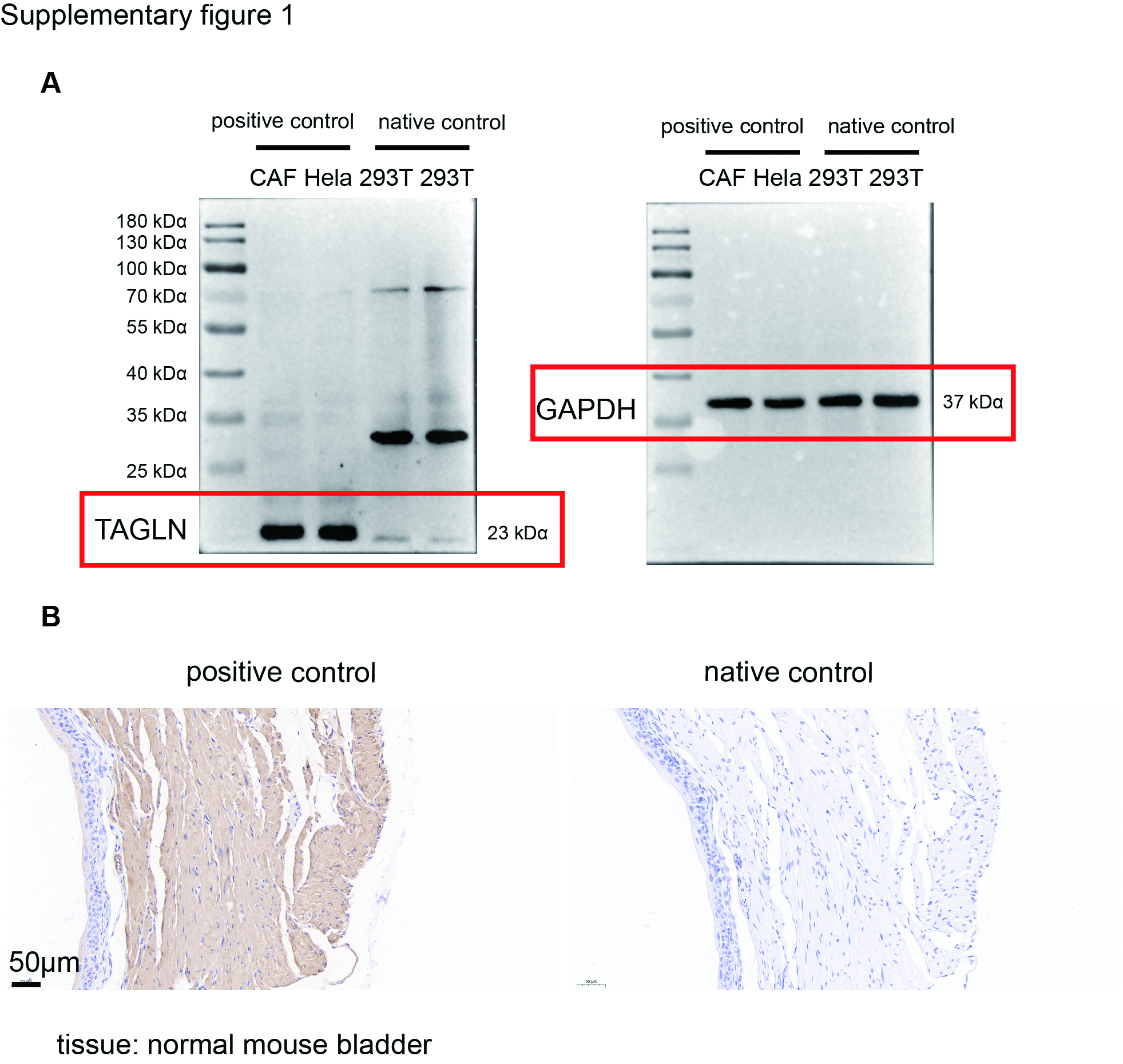

Supplement: Supplementary file 2 — supplementary figure 1 [file 41389_2023_463_MOESM2_ESM.tif]

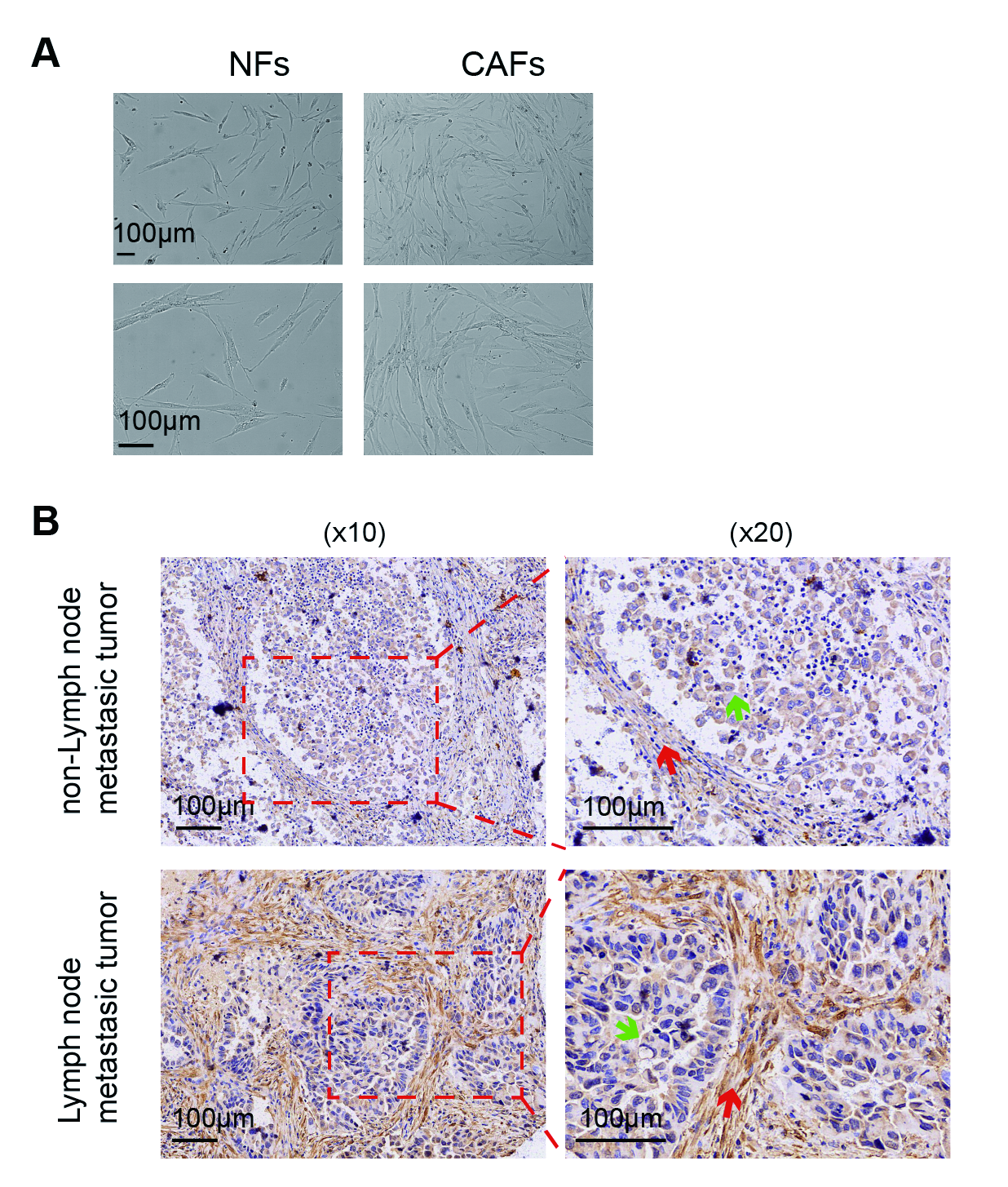

Supplement: Supplementary file 3 — supplementary figure 2 [file 41389_2023_463_MOESM3_ESM.tif]

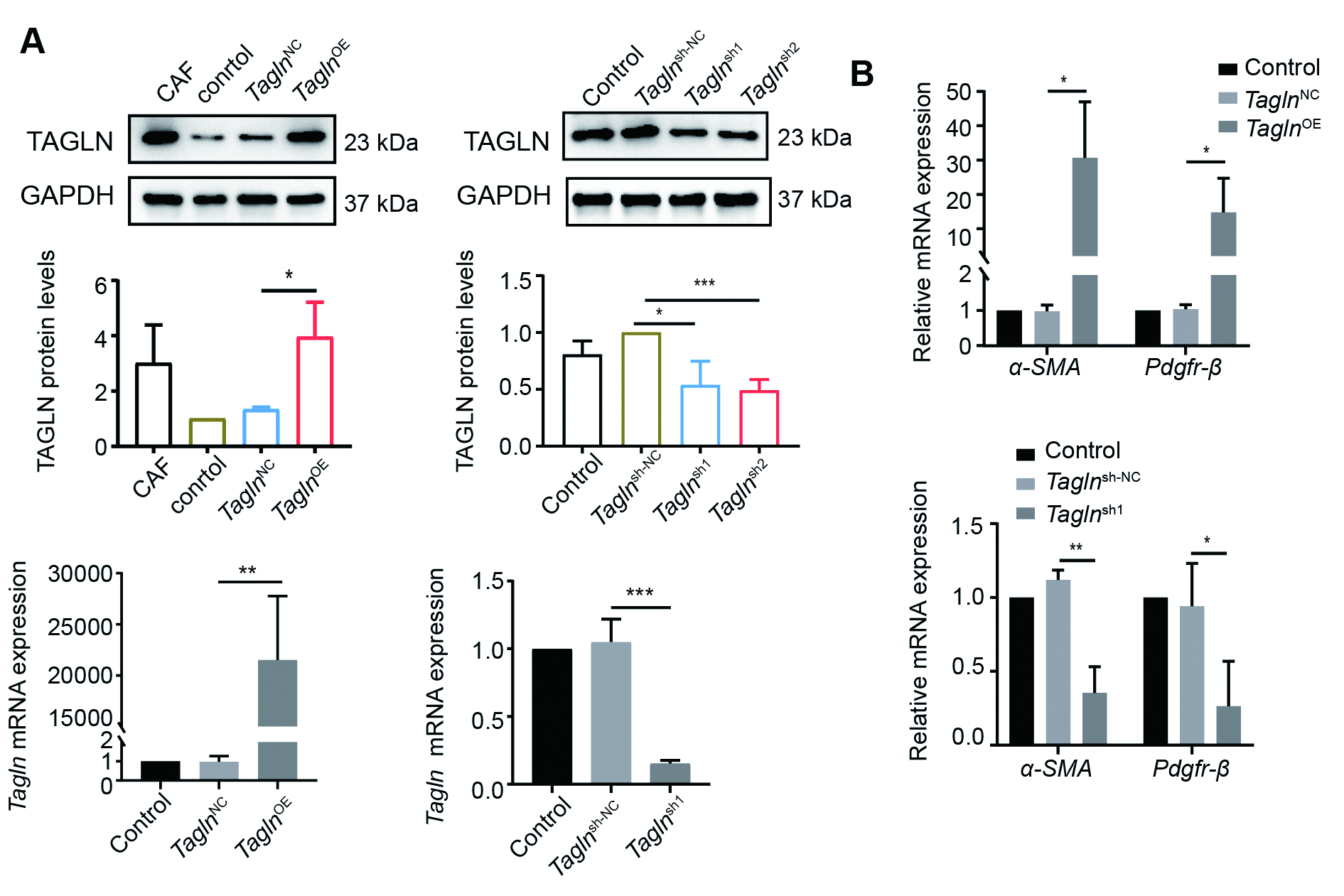

Supplement: Supplementary file 4 — supplementary figure 3 [file 41389_2023_463_MOESM4_ESM.tif]

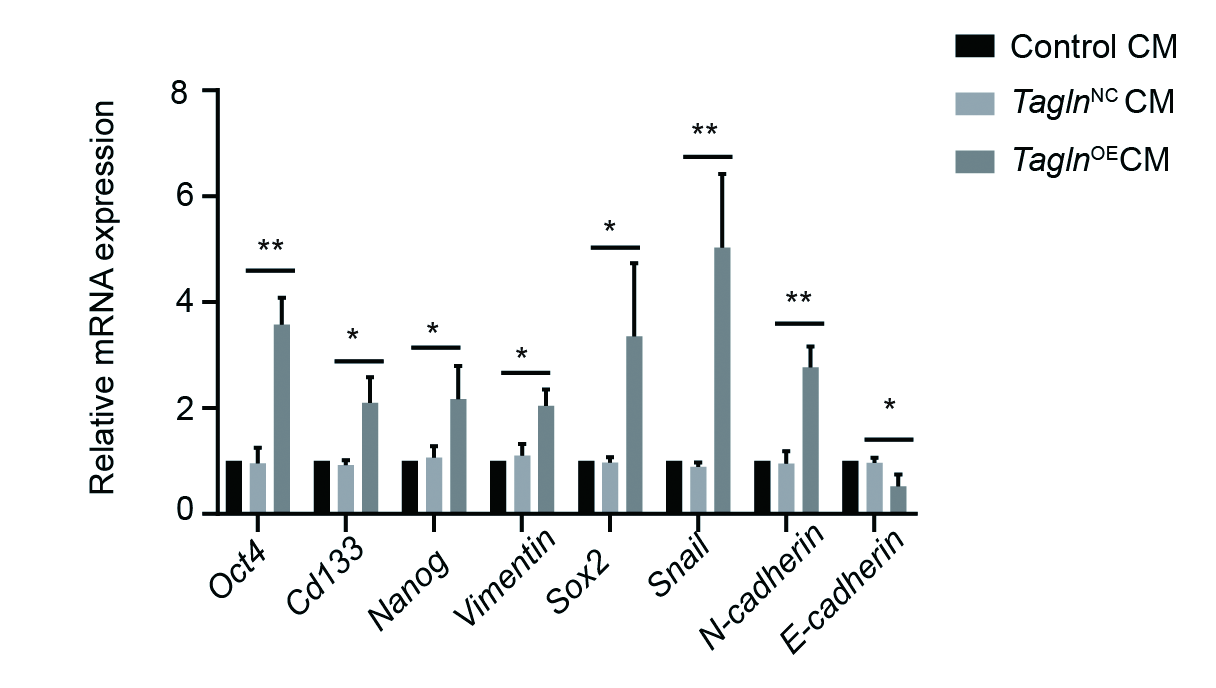

Supplement: Supplementary file 5 — supplementary figure 4 [file 41389_2023_463_MOESM5_ESM.tif]

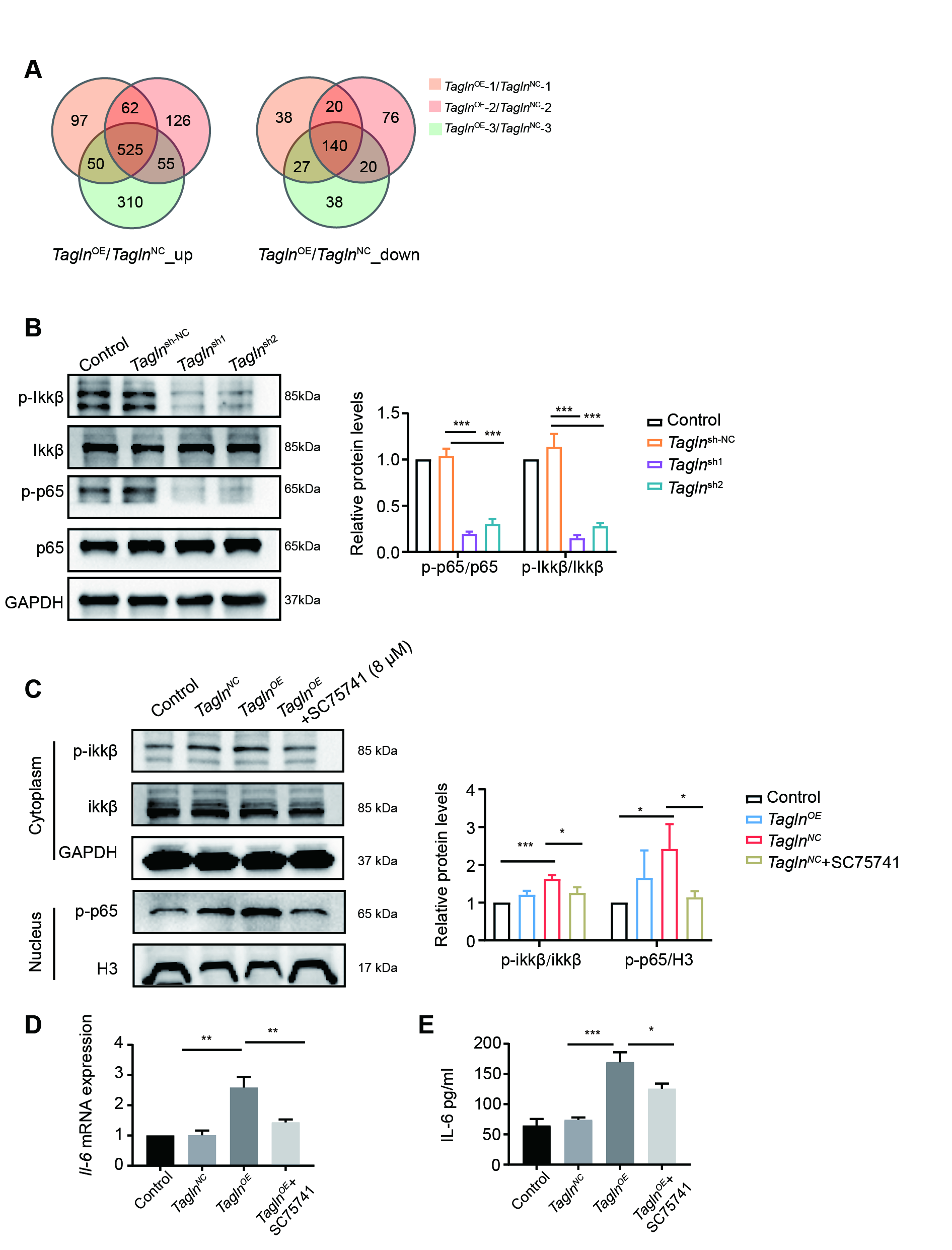

Supplement: Supplementary file 6 — supplementary figure 5 [file 41389_2023_463_MOESM6_ESM.tif]

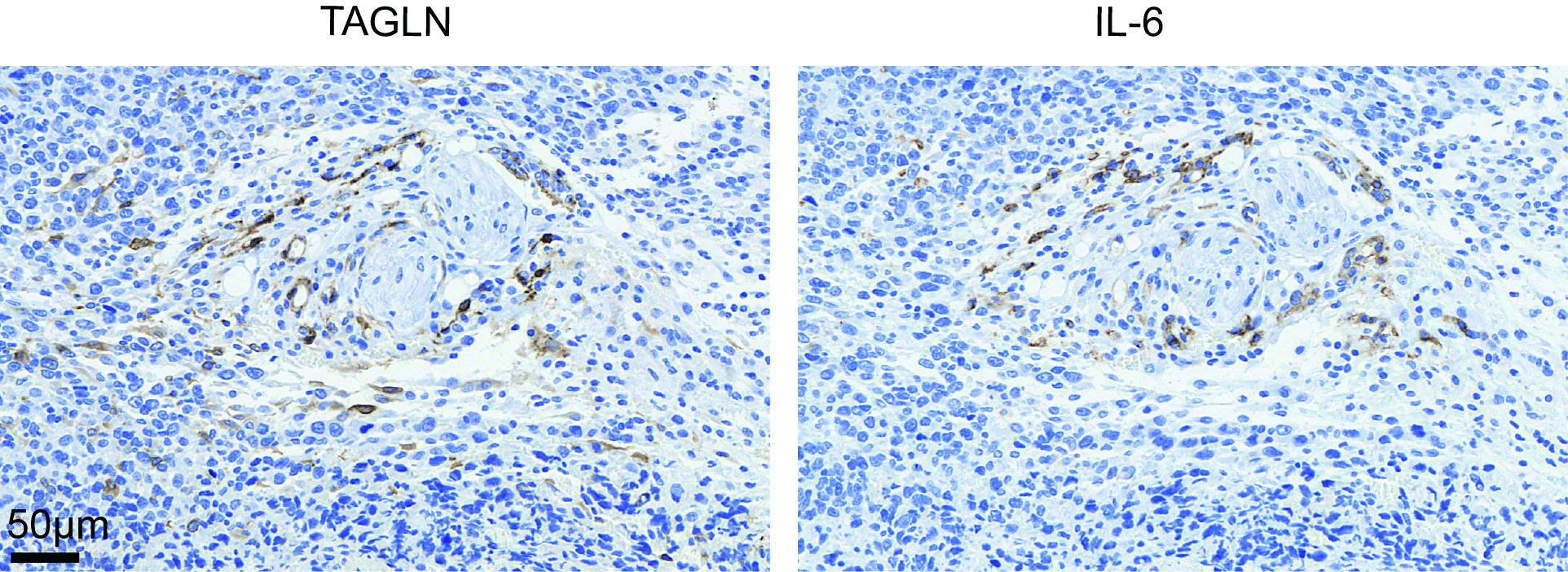

Supplement: Supplementary file 7 — supplementary figure 6 [file 41389_2023_463_MOESM7_ESM.tif]
