# Supplementary material for: Transgelin promotes lung cancer progression via activation of cancer-associated fibroblasts with enhanced IL-6 release
Source: Oncogenesis. 2023 Mar 29;12(1):18. doi: 10.1038/s41389-023-00463-5 (PMC10060230; doi:10.1038/s41389-023-00463-5)
Supplement: Supplementary file 8 — Supplementary Table 1 [file 41389_2023_463_MOESM8_ESM.docx]

**Table S1 List of primers sequences.**

| Primer name | Forward primer (5′–3′) | Reverse primer (5′–3′) |
| --- | --- | --- |
| *Tagln* | CAACAAGGGTCCATCCTACGG | ATCTGGGCGGCCTACATCA |
| *Gapdh* | TGGCCTTCCGTGTTCCTAC | GAGTTGCTGTTGAAGTCGCA |
| *α-SMA* | GTCCCAGACATCAGGGAGTAA | TCGGATACTTCAGCGTCAGGA |
| *Pdgfr-β* | TTCCAGGAGTGATACCAGCTT | AGGGGGCGTGATGACTAGG |
| *Il-6* | TAGTCCTTCCTACCCCAATTTCC | TTGGTCCTTAGCCACTCCTTC |
| *Oct4* | GGCTTCAGACTTCGCCTCC | AACCTGAGGTCCACAGTATGC |
| *Sox2* | GCGGAGTGGAAACTTTTGTCC | CGGGAAGCGTGTACTTATCCTT |
| *Cd133* | CTCCCATCAGTGGATAGAGAACT | ATACCCCCTTTTGACGAGGCT |
| *Nanog* | TCTTCCTGGTCCCCACAGTTT | GCAAGAATAGTTCTCGGGATGAA |
| *E-cadherin* | CAGGTCTCCTCATGGCTTTGC | CTTCCGAAAAGAAGGCTGTCC |
| *Vimentin* | CGTCCACACGCACCTACAG | GGGGGATGAGGAATAGAGGCT |
| *N-cadherin* | AGCGCAGTCTTACCGAAGG | TCGCTGCTTTCATACTGAACTTT |
| *Snail* | CACACGCTGCCTTGTGTCT | GGTCAGCAAAAGCACGGTT |
| *18s* | ACCGCAGCTAGGAATAATGGA | CAAATGCTTTCGCTCTGGTC |

All primers are mouse-derived
